# Minimum Criteria for DNA Damage-Induced Phase Advances in Circadian Rhythms

**DOI:** 10.1371/journal.pcbi.1000384

**Published:** 2009-05-08

**Authors:** Christian I. Hong, Judit Zámborszky, Attila Csikász-Nagy

**Affiliations:** 1 Department of Genetics, Dartmouth Medical School, Hanover, New Hampshire, United States of America; 2 The Microsoft Research–University of Trento Centre for Computational and Systems Biology, Povo (Trento), Italy; University of Washington, United States of America

## Abstract

Robust oscillatory behaviors are common features of circadian and cell cycle rhythms. These cyclic processes, however, behave distinctively in terms of their periods and phases in response to external influences such as light, temperature, nutrients, etc. Nevertheless, several links have been found between these two oscillators. Cell division cycles gated by the circadian clock have been observed since the late 1950s. On the other hand, ionizing radiation (IR) treatments cause cells to undergo a DNA damage response, which leads to phase shifts (mostly advances) in circadian rhythms. Circadian gating of the cell cycle can be attributed to the cell cycle inhibitor kinase Wee1 (which is regulated by the heterodimeric circadian clock transcription factor, BMAL1/CLK), and possibly in conjunction with other cell cycle components that are known to be regulated by the circadian clock (i.e., c-Myc and cyclin D1). It has also been shown that DNA damage-induced activation of the cell cycle regulator, Chk2, leads to phosphorylation and destruction of a circadian clock component (i.e., PER1 in *Mus* or FRQ in *Neurospora crassa*). However, the molecular mechanism underlying how DNA damage causes predominantly phase advances in the circadian clock remains unknown. In order to address this question, we employ mathematical modeling to simulate different phase response curves (PRCs) from either dexamethasone (Dex) or IR treatment experiments. Dex is known to synchronize circadian rhythms in cell culture and may generate both phase advances and delays. We observe unique phase responses with minimum delays of the circadian clock upon DNA damage when two criteria are met: (1) existence of an autocatalytic positive feedback mechanism in addition to the time-delayed negative feedback loop in the clock system and (2) Chk2-dependent phosphorylation and degradation of PERs that are not bound to BMAL1/CLK.

## Author Summary

Molecular components and mechanisms that connect cell cycle and circadian rhythms are important for the well-being of an organism. Cell cycle machinery regulates the progress of cell growth and division while the circadian rhythm network generates an ∼24 h time-keeping mechanism that regulates the daily processes of an organism (i.e. metabolism, bowel movements, body temperature, etc.). It is observed that cell divisions usually occur during a certain time window of a day, which indicated that there are circadian-gated cell divisions. Moreover, it's been shown that mice are more prone to develop cancer when certain clock genes are mutated resulting in an arrhythmic clock. Recently, a cell cycle checkpoint regulator, Chk2, was identified as a component that influences a core clock component and creates mostly phase advances (i.e., jet lags due to traveling east) in circadian rhythms upon DNA damage. This phase response with minimum delays is an unexpected result, and the molecular mechanism behind this phenomenon remains unknown. Our computational analyses of a mathematical model reveal two molecular criteria that account for the experimentally observed phase responses of the circadian clock upon DNA damage. These results demonstrate how circadian clock regulation by cell cycle checkpoint controllers provides another layer of complexity for efficient DNA damage responses.

## Introduction

Circadian rhythms are periodic physiological events that recur about every 24 hours. The importance of circadian rhythms is well recognized in many different organisms' survival as well as in human physiology. Misregulations in circadian rhythms may lead to different conditions such as depression, familial advanced sleep phase syndrome (FASPS), delayed sleep phase syndrome (DSPS), or insomnia, which largely impact our society [1],[2]. Recent studies indicate higher incidents of cancer in clock defective individuals [3],[4] and chronic jet-lag is associated with higher mortality rate in aged mice as well as faster growth of tumor [5],[6]

The molecular mechanism of circadian rhythms began to become clear beginning with the discovery of the *period* (*per*) gene in *Drosophila melanogaster* in 1971 [7], and the *frequency* (*frq*) gene in *Neurospora crassa* in 1973 [8]. Through analysis of the genetic variants of these genes, pieces of the clock's mechanism could be described. The consensus idea is that it involves interlocked feedback loops largely based on a transcription-translation related time-delayed negative feedback loop [9]. Most of the genes encoding proteins involved in the mechanism of circadian rhythms have been found simply by screens aimed at cataloging the components or by analysis of the regulation of the components. Several studies of mathematical modeling and systems approaches helped further understanding of circadian rhythms in various organisms [10]–[14].

One of the defining properties of circadian rhythms is the ability to phase shift upon a stimulus from external cues. This property allows organisms to adapt efficiently to the external environment. For example, a person traveling east to Europe from the U.S. will experience a jet-lag in the process to adapt advanced phase. Even a brief pulse of light may cause phase advances or delays depending on the timing and influence of the pulse [15]. It is intuitive to assume that a phase shifting agent will create both phase advances and delays depending on the timing and strength of the pulse by uniformly affecting molecular pathways in the circadian system [16]. It has been observed that 2 h treatments of Rat-1 fibroblasts with dexamethasone (Dex) result in large advances and delays (Type 0 resetting of the phase), possibly by inducing transcription of both *rPer1* and *rPer2* [17],[18]. This Dex-dependent PRC is also observed in the NIH3T3-Bmal1-Luc-1 cells [19]. If the Dex-dependent induction of *Per* transcripts causes both phase advances and delays, we would also predict that DNA damage-dependent phosphorylation and degradation of PERs by Chk2 [20],[21] would result in similar PRCs. Recent findings indicate that this prediction is wrong [18],[21]. Upon experiencing DNA damage, the cell cycle machinery influences the circadian clock in such a way that creates predominantly phase advances in Rat-1 fibroblasts and mice [18], as well as in *Neurospora crassa* [21]. These data strongly suggest that there is a conserved pathway across different species that affects the phase of the clock after DNA damage, and involves physical interactions of ATM and/or Chk2 with a core clock component (i.e. PER1 or FRQ) [18],[20],[21]. This interaction leads to phosphorylation of PER1 and FRQ [21],[22]. The molecular mechanism for this unique phenomenon, however, remains unexplained.

In this paper, we explore the minimum criteria in the molecular network of circadian rhythms that simulate the above PRCs with tools of computational modeling. Theoretically, a time-delayed negative feedback is sufficient to create robust oscillations. Both cell cycle and circadian rhythms, however, contain both negative and positive feedbacks in their wiring networks. Positive feedback mechanisms are essential for proper eukaryotic cell divisions [23] whereas their roles in circadian rhythms remain elusive. Recently, Tsai and colleagues indicated that a general function of positive feedbacks in different networks is to create tunable robustness in the system [24]. In our study, we address two questions 1) what is a molecular mechanism that accounts for Chk2-dependent PRC in circadian rhythms?, and 2) is the positive feedback mechanism necessary for the observed PRC? In the conditions that we have tested, we discovered that we can only simulate the Chk2-dependent PRC with predominantly phase advances when Chk2 only affects PERs that are not bound to BMAL1/CLK in the presence of an autocatalytic positive feedback mechanism. Both conditions are required for proper simulations. Our study is the only in silico experiment to indicate the necessity of an autocatalytic positive feedback mechanism in simulating specific phenotype in the circadian system.

## Results

### Chk2-dependent differential degradation of PER creates predominantly phase advances upon DNA damage

**Figure 1:**
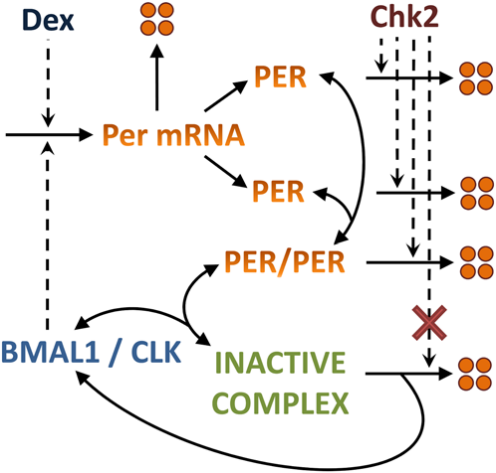
Molecular wiring diagram of the simple circadian clock network. For simplicity of the model, we only deal with PER protein, and treat PER1, PER2, and PER3 as same proteins. We assume that PERs exist in monomers, dimers, and complex with the BMAL1/CLK. We also assume that the BMAL1/CLK is inactive when bound to PER forming a negative feedback loop. A pulse of Dex activates the transcription of *Per* in addition to the BMAL1/CLK. Chk2 does not affect the PERs that are bound to the BMAL1/CLK, which accounts for the unique phase response upon DNA damage.

**Figure 2:**
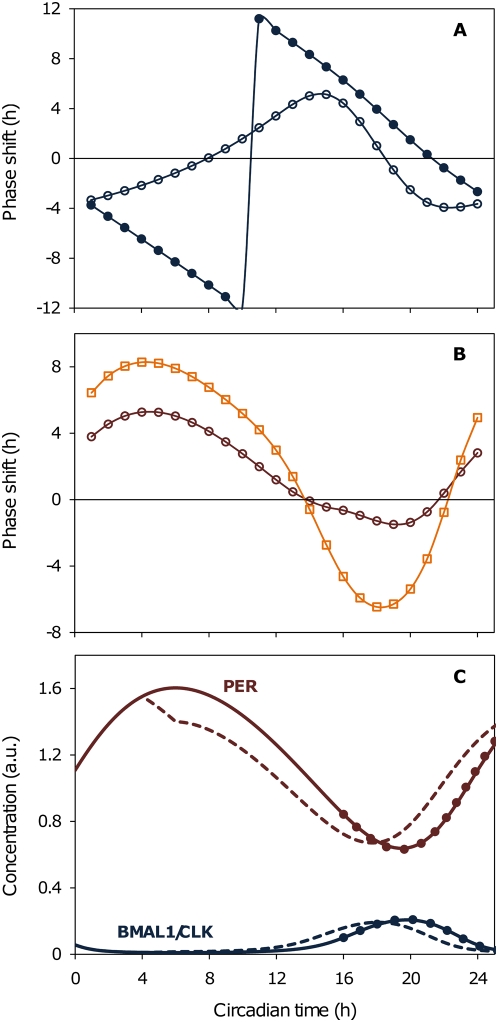
In silico Dex and IR treated experiments. (A) Strong pulses of Dex generate Type 0 PRC (filled circles; strong resetting of the circadian clock to the new phase which does not depend on the old phase) whereas weak pulses of Dex generates Type 1 PRC (blank circles; weak resetting of the phase where the new phase changes as a function of the old phase). (B) Large advances and delays are observed when Chk2 is assumed to affect all forms of PERs including the complex with BMAL1/CLK (orange squares). Chk2-dependent phase advances and minimum delays of the circadian clock are observed only if Chk2 does not affect the PERs that are in complex with BMAL1/CLK (red circles). (C) DNA damage-induced Chk2 activation causes phase advances of circadian clock. Solid lines represent endogenous profiles of PER and BMAL1/CLK. Dashed lines indicate PER (red - *CP_total_*) and BMAL1/CLK (blue - *TF*) in response to a 2 h IR treatment at simulation hour 4 and dots represent the results after the same 2 hr treatment at hour 16 (hour 0 corresponds to the peak of PER monomers (*CP*)).

We explored our simple mammalian circadian clock model (Fig. 1) from our previous work [25] to investigate whether we can simulate different PRCs from the Dex and IR treatment experiments [17],[18]. Note that an autocatalytic positive feedback mechanism is already embedded in our model [12],[26]. Based on the experimental data, we added the following in our previous model: 1) Dex increases the transcripts of *Per* but not *Bmal1* [18], and 2) Chk2 phosphorylates PERs and facilitates their degradation upon DNA damage [20],[21]. Our simulations show that the Dex-dependent increase of *Per* messages creates both Type 0 (as shown in the experiment, strong resetting of the phase) and Type 1 PRCs (weak resetting of the phase) depending on the strength (concentration) of the Dex treatments (Fig. 2A). It is, however, not trivial to simulate a PRC with mostly phase advances reproducing the phenotype from the IR treatment experiments [18]. We observe a PRC with large advances and delays if we follow the simplest possible assumption that DNA damage induces Chk2-dependent phosphorylation and degradation of all forms of PER (monomer, dimer, and complex with BMAL1/CLK) (Fig 1 and Fig 2B). Through in silico experiments, however, we observe minimum phase delays as seen in experiments [18],[21] only when Chk2 does not affect the PER that is in a complex with BMAL1/CLK (i.e. due to conformational changes of PER upon complex formation) (Fig. 2B). In other words, Chk2 prematurely degrades PERs that are not bound to BMAL1/CLK to advance the clock, while allowing continued repression of BMAL1/CLK by not degrading the PERs that are in complex with BMAL1/CLK (Fig. 2C). This prolonged repression on BMAL1/CLK creates small delays when Chk2 affects PERs around their minima as observed in experiments [18],[21].

It is interesting to note that an inhibition of CKIε, another kinase that is known to phosphorylate PER, generates a PRC with only delays [27]. This PRC is qualitatively different than the PRC after DNA damage as there are no advances. We can simulate a mirror image of the PRC with mostly advances, which creates mostly delays, by reducing the rates for Chk2-dependent phosphorylations (not shown). Our data, however, is qualitatively different as we do see small advances whereas Badura and colleagues did not observe any advances [27]. This difference are possibly due to the following reasons: 1) Badura et al. administered a CKIε inhibitor not as a pulse (there was no removal of the drug after administration), and 2) it is possible that Chk2 and CKIε results in different types of phosphorylations which can lead to different consequences. We plan to further investigate this with an extended version of circadian clock module.

### An autocatalytic positive feedback mechanism is required for the observed PRC

Our simple model is adapted from Tyson and colleagues' earlier paper where both negative and positive feedbacks play essential roles in creating a robust oscillator [12],[26]. The autocatalytic positive feedback mechanism in the model arises from different stabilities between PER monomers vs. PER complexes. Based on molecular data from *Drosophila* system [28]–[31], we assume that PER monomers are more susceptible to degradation than PER in complexes (i.e. PER/PER, PER/CRY, etc.). This creates autocatalytic PER dynamics as PER stabilizes itself by forming complexes. To date, this is the only circadian rhythm model that employs an essential positive feedback mechanism that is necessary to maintain a robust oscillator [32]. Hence, we wondered whether the incorporated essential positive feedback is required (or disposable) in simulating the unique PRCs upon DNA damage.

In order to test our hypothesis, we removed the autocatalysis in the model by assuming no stability differences between PER monomers and complexes. Then, we re-parameterized the system to rescue oscillations (see materials and methods). Note that we had to use a Hill-coefficient = 4 for highly cooperative negative feedback in order to rescue oscillations in our four-variable model in the absence of the autocatalytic positive feedback mechanism. To our surprise, we were not able to generate the unique PRC with predominantly phase advances upon DNA damage even by assuming differential phosphorylation and degradation of PER monomers vs. PER complexes with BMAL1/CLK (lane 2, Table 1).

**Table 1:** Theoretical requirements for the experimentally observed DNA damage-induced PRCs with small delays in circadian clock models.

Model	Positive feedback	Ratio of maximum advance and maximum delay
Simple model	Yes	3.54
Simple model, positive feedback removed	No	0.77
Leloup and Goldbeter set 1	No	0.57
Leloup and Goldbeter set 3	No	1.11
Leloup and Goldbeter set 1 with positive feedback	Yes	0.71
Leloup and Goldbeter set 3 with positive feedback	Yes	2.47

We removed the autocatalytic positive feedback from our simple model and added positive feedback into the Leloup and Goldbeter's model as discussed in the text. In all cases, we checked the maxima and minima from PRCs after the Chk2-dependent degradations of PER. In the last column, we report the ratio of these values (larger value indicates most advance with least delay). See text for analysis and Table S1 for detailed results. In all cases we assume that Chk2 acts only on the free forms of PER.

We wondered whether above conclusions from our simple model can be generalized to a more comprehensive model with distinct wiring network. Hence, we tested Leloup and Goldbeter's mammalian model [33],[34]. They used four sets of parameters in order to investigate possible functions of multiple feedback loops in the circadian system. For our purposes, we concentrated in parameter sets 1 and 3. In the parameter set 1, robust oscillations of their model can arise from two different time-delayed negative feedback loops: PER-driven and PER/CRY-independent BMAL1/CLK-driven negative feedback loops. For this parameter set, they can generate an oscillator based on BMAL1/CLK-driven negative feedback loop in the absence of the PER-driven negative feedback loop. In the parameter set 3, they disabled the BMAL1/CLK-driven negative feedback loop making the system a PER/CRY-dependent single negative feedback oscillator. We did not explore parameter sets 2 and 4 because PER is not required for oscillations in parameter sets 2 and 4. The wiring network of Leloup and Goldbeter's model is significantly different from our model which consists of an intertwined dynamics between an essential autocatalytic positive feedback and time-delayed negative feedback [12],[32].

We incorporated Chk2-induced degradation of PER molecules that are not bound to BMAL1/CLK in the Leloup and Goldbeter's model. Then, we tested Chk-2-dependent differential degradation of PER as in our simple model. Our simulations indicate that we see both TYPE 1 and TYPE 0 PRC depending on the strength of Chk2, but we do not observe asymmetric PRCs with mostly advances (lane 3 and 4, Table 1). These results show that the differential effect of Chk2-dependent degradation of PER complexes is not enough to create the observed DNA-damage induced PRCs with the innate wiring of the Leloup and Goldbeter's model.

Our next step was to introduce an autocatalytic positive feedback mechanism in the Leloup and Goldbeter's model and investigate its role in reproducing the asymmetric PRC upon DNA-damage. First, we added an autocatalytic positive feedback in the parameter set 1 of Leloup and Goldbeter's model in a similar way as in our simple model. PER complexes are assumed to be more stable than PER monomers. To our surprise, we were not able to generate the PRCs with predominantly phase advances with differential degradations of PER complexes by Chk2 even with an added autocatalytic positive feedback mechanism (lane 5, Table 1). We wondered whether this was due to the PER-independent BMAL1/CLK-driven negative feedback loop which is built in the parameter set 1. Hence, we tested the parameter set 3 which consists of the PER-driven single negative feedback. Interestingly, we were able to simulate the observed asymmetric PRC with predominantly phase advances as we have observed in our simple model only when both the autocatalytic positive feedback and the differential effect of Chk2 on PERs were implemented in the absence of BMAL1/CLK-driven negative feedback loop (lane 6, Table 1). This suggests that there exists an important dynamical relationship between negative feedback loops and an autocatalytic positive feedback mechanism.

## Discussion

What are the implications of DNA damage-induced phase responses of the circadian clock to the cell cycle? We hypothesize that cells utilize various pathways for different timing events in response to DNA damage. The Chk2 kinase directly inhibits the progress of the cell cycle by phosphorylating and removing Cdc25C (a phosphatase that is antagonistic to Wee1 which activates cell proliferation) from the nucleus [35]. Moreover, the cell cycle machinery also employs Chk2 in order to provide an additional mechanism that helps to delay the cell cycle progress for extended time by indirectly increasing the level of Wee1 via the circadian network. We believe that the above sequential roles of Chk2 maximize the efficiency of DNA damage-induced delay. With our model, we show that premature degradation of PER, resulting in phase advances, causes early activation of BMAL1 (Fig 2C). This creates an early transcriptional activation of the Wee1 (G2 inhibitor of the cell cycle) during the upcoming circadian cycle, which delays the cell cycle in the G2 phase. If the DNA damage-response induces large phase delays, it will generate a short-lived, transient increase of BMAL1, but a long delay in the activation of Wee1 by BMAL1/CLK for the upcoming circadian cycle. This late activation of Wee1 is probably not a desired result for an efficient DNA damage response.

Our model is simple and intuitive, and yet predicts a molecular mechanism that is responsible for the observed PRC. Our in silico experiments elucidate a molecular mechanism that accounts for Chk2-dependent phase advances and minimum delays of the circadian clock upon DNA damage. It seems counterintuitive to assume that Chk2 does not affect the PER that is in a complex with BMAL1/CLK. This may appear to prolong the repression on BMAL1, which will delay the activation of Wee1. However, due to the cyclic nature of the circadian clock, our simulations suggest that these unique Chk2-dependent phase responses are the best strategy for inducing large and prolonged induction of Wee1 by BMAL1/CLK, allowing extended time for the cell cycle to repair problems upon DNA damage. We propose that the cell cycle network is ingeniously wired with the circadian clock for an optimal response upon DNA damage. Previously, experimentalists showed that the functional circadian clock is important for optimum response to the chemotherapeutic agent cyclophosphamide or γ radiation [4],[36]. For example, reduced apoptosis is observed in m*Per2* deficient mice compared to wild-type mice upon γ radiation, which resulted in tumorigenesis [4]. Based on these works, it can be assumed that DNA damage response is more efficient when the circadian clock is intact. We do not know, however, how the efficiency of DNA damage response is affected by the circadian clock. Hence, we suggest testing the efficiency of DNA damage response in the presence and absence of the circadian clock in both in cell culture (i.e. wild-type vs. *cry^ko^*) as well as in vivo.

Another intriguing finding is the importance of the autocatalytic positive feedback mechanism in simulating the observed PRC upon DNA damage. Our simple model is adapted from Tyson and colleagues which implemented both negative and positive feedback mechanisms [12],[32]. DNA damage-induced PRCs with predominantly advances are lost upon removal of the positive feedback even with the differential degradation of PERs by Chk2. This observation is extended to the Leloup and Goldbeter's model [33],[34]. We tested four different combinations of positive and negative feedback loops with two different sets of parameters (Table 1). Our findings confirm that the autocatalytic positive feedback mechanism is required to simulate DNA damage-induced PRCs. Our results elucidate three important points: (1) the role of the autocatalytic positive mechanism in the circadian system, (2) the wiring of different negative feedback loops, and (3) the interplay between positive and negative feedbacks in response to DNA damage. We acknowledge that there are multiple feedback loops in the circadian system [9]. Therefore, it is essential to develop a more comprehensive model accounting detailed dynamics of different negative feedback loops in the clock network. Furthermore, it is important to experimentally verify autocatalytic positive feedback mechanisms in the context of circadian rhythms, the nonlinearity of negative feedback loops, and the possible interplay between the positive and negative feedback loops in the circadian clock.

## Materials and Methods

### Circadian rhythm model

Our objective is to create a simple mammalian circadian clock model that accounts for different phase response curves (PRCs) observed from various experiments [17],[18],[21]. For simplicity of the model, we only deal with PER protein and treat PER1, PER2, and PER3 as same proteins. CRY proteins (CRY1 and CRY2) are also part of core clock components that negatively regulate BMAL1/CLK. We do not consider, however, CRY proteins in this model for two reasons: (1) simplicity of the model, and (2) it is not yet known whether Chk2 phosphorylates and triggers degradation of CRY proteins as mPER1. We will include the function of CRY proteins in our future work. We assume that PERs exist in monomers (Clock Protein, CP), dimers (Clock Protein, CP_2_), and complex with the BMAL1/CLK (Transcription Factor, TF). We imagine that the BMAL1/CLK is inactive when bound to PER (Inactive Complex, IC) creating a negative feedback. We treat CLK as a parameter in the system since it does not cycle [37]. We also assume that the CP_2_ is more stable than the CP, which introduces a positive feedback in the system [12]. Dex induces the transcription of *Per* message (Message, M) [18], and DNA damage-activated Chk2 promotes phosphorylation and degradation of PERs [20],[21]. We use same equations and parameter values from our previous publication [25] other than the newly added effects of Dex or Chk2.

### Differential equations of the simplified circadian rhythm model for mammalian cells

Messenger RNA of the clock proteins (*Per* mRNA):
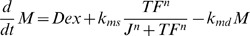
(1)Monomer clock proteins (PER):
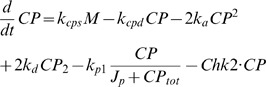
(2)Dimer form of clock proteins (PER/PER):
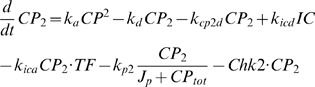
(3)Transcription factor (BMAL1/CLK):
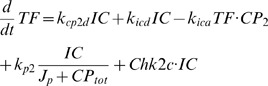
(4)Inactive complex of clock dimers and transcription factor:
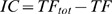
(5)Total amount of clock proteins (PER on Fig. 2):
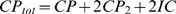
(6)Rate constants (h^−1^):
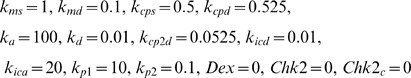
Dimensionless constants:
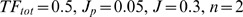



All protein concentrations in the model are expressed in arbitrary units (au) because, for the most part, we do not know the actual concentrations of most circadian proteins in the cell. All rate constants capture only the timescales of processes (rate constant units are in h^−1^).

### Simulation of Dex and IR treatments

1. Strong resetting (type 0 PRC) of circadian period by Dex treatment (2 h pulse):
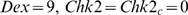

2. Weak resetting (type 1 PRC) of circadian period by Dex treatment (2 h pulse):
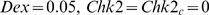

3. Chk2 affects degradation of all forms of PER, including inactive complex (*IC*) of transcription factor BMAL1/CLK (*TF*) and PER dimers (2 h treatment).
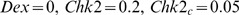

4. Chk2 only affects degradation of PER monomers and dimers (2 h treatment).
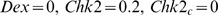

### Removal of the positive feedback mechanism from Zámborszky et al. [25]

Various parameters of the model of Zámborszky et al. [25] have been changed in order to remove the originally existing positive feedback from the system. The equations are the same as presented above. Many parameters were changed to create a robust circadian rhythm with approx 24 h period. Changed parameters: Rate constants (h-1): *k_ms_* = 0.5, *k_md_* = 0.045, *k_cps_* = 10, *k_cpd_* = 0.0001, *k_a_* = 100, *k_d_* = 0.001, *k_cp2d_* = 0.0001, *k_icd_* = 0.001, *k_ica_* = 4, *k_p1_* = 1.97, *k_p2_* = 1.97. Dimensionless constants: *TF_tot_* = 1, *J_p_* = 0.05, *J* = 0.4, *n* = 4.

### Simulation of IR treatments in the Leloup and Goldbeter's model [33],[34]

The Chk2 induces degradation of PER monomers and PER-CRY dimers but not PER proteins that are in complex with BMAL1/CLK. To achieve this we replaced the original *V_phos_* term by (*V_phos_*+*V_Chk2_*) in the original Leloup and Goldbeter models [33],[34]. In simulations we used *V_Chk2_* = 1 to simulate the effect of IR pulse treatment.

### Addition of a positive feedback mechanism to the Leloup and Goldbeter's model [33],[34]

We increased the nonspecific degradation rate constant for destruction of nonphosphorylated PER monomers in the cytosol from 0.01 to 0.3, while keeping the background degradation rates of PER/PER dimers and PER/CRY complexes at the original 0.01 level. In this way PER has a positive influence on itself by forming complexes. This creates a similar autocatalytic positive feedback mechanism as the one we used in Zámborszky et al. [25].

### Computer simulations

We used XPP-AUT computer program [38] of G. Bard Ermentrout (freely available at http://www.math.pitt.edu/~bard/xpp/xpp.html) for simulations and analysis of our model. The ODE file of our model is available as online supplementary material of this article (see Text S1). The SBML version of the model is also downloadable from the BioModels Database (http://www.ebi.ac.uk/biomodels-main/) [39], as MODEL7984093336. For each simulation, we calculated the phase differences between unperturbed and perturbed systems after 10 days (10 circadian cycles). Treatments were induced at each circadian hour.

## Supporting Information

Table S1 Detailed results of the positive feedback necessity analysis of Table 1.
(0.03 MB DOC)

Text S1 Readers can simulate this model by the XPP-AUT computer program, freely available at http://www.math.pitt.edu/~bard/xpp/xpp.html
(0.00 MB TXT)
