# Supplementary material for: Minimum Criteria for DNA Damage-Induced Phase Advances in Circadian Rhythms
Source: PLoS Comput Biol. 2009 May 8;5(5):e1000384. doi: 10.1371/journal.pcbi.1000384 (PMC2677641; doi:10.1371/journal.pcbi.1000384)
Supplement: Table S1 — Detailed results of the positive feedback necessity analysis of Table 1. (0.03 MB DOC) [file pcbi.1000384.s001.doc]

**Table S1. Detailed results of the positive feedback necessity analysis of Table 1**

| MODEL | Circadian period | Changed parameters | Chk2 value | Maximum advance (h) | Maximum delay  (h) | Ratio of maximum advance and maximum delay |
| --- | --- | --- | --- | --- | --- | --- |
| *Simple model* | 24.0 | - | 0.2 | 5.27 | -1.49 | 3.54 |
| *Simple model*,  positive feedback removed | 26.8 | many^[1]^ | 0.05 | 8.89 | -11.56 | 0.77 |
| *Leloup and Goldbeter Set1* | 24.0 | - | 1 | 1.24 | -2.18 | 0.57 |
| *Leloup and Goldbeter Set 3* | 23.9 | - | 1 | 3.94 | -3.55 | 1.11 |
| *Leloup and Goldbeter Set1*  with positive feedback | 25.2 | *k_dnp_* = 0.3^[2]^ | 1 | 1.69 | -2.39 | 0.71 |
| *Leloup and Goldbeter*  Set 3  with positive feedback | 20.8 | *k_dnp_* = 0.3^[2]^ | 1 | 10.66 | -4.32 | 2.47 |

^[1]^: Parameter set (without positive feedback):

Rate constants (h^-1^): *k_ms_* = 0.5, *k_md_* = 0.045, *k_cps_* = 10, *k_cpd_* = 0.0001, *k_a_* = 100, *k_d_* = 0.001, *k_cp2d_* = 0.0001, *k_icd_* = 0.001, *k_ica_* = 4, *k_p1_* = 1.97, *k_p2_* = 1.97. Dimensionless constants: *TF_tot_* = 1, *J_p_* = 0.05, *J* = 0.4, *n* = 4.

^[2]^: We introduced a new rate constant *k_dnp_* as the nonspecific degradation rate constant of nonphosphorylated PER proteins in the cytosol (*P_C_* in their model).
